# Molecular regulation of vascular endothelial growth factor expression in the retinal pigment epithelium

**Published:** 2012-03-01

**Authors:** Knatokie M. Ford, Patricia A. D’Amore

**Affiliations:** 1 Schepens Eye Research Insititute/Massachusetts Eye and Ear, Boston, MA; 2 Program in Biological and Biomedical Sciences, Harvard Medical School, Boston, MA; 3 Department of Ophthalmology, Harvard Medical School, Boston, MA; 4 Department of Pathology, Harvard Medical School, Boston, MA

## Abstract

**Purpose:**

Vascular endothelial growth factor (VEGF) plays an important role in homeostasis and diseases of the retinal pigment epithelium (RPE), choriocapillaris, and, most notably, age-related macular degeneration (AMD). Although much is known about VEGF regulation in pathologies, little is known about the control of VEGF expression under normal conditions. VEGF expression has been previously shown to be regulated in coordination with cell differentiation in the muscle and kidney. We therefore tested the hypothesis that VEGF in the adult RPE would similarly be regulated in conjunction with differentiation.

**Methods:**

A human retinal pigment epithelium cell line (ARPE-19), a line of immortalized human RPE cells, was used for all experiments. RPE cells were polarized in culture for 4 weeks on laminin-coated Transwells. Levels of *VEGF* mRNA and protein were determined with real-time PCR and enzyme-linked immunosorbent assay, respectively. *VEGF*-luciferase reporter constructs were used to identify regions of the *VEGF* promoter that control *VEGF* expression in the RPE. Microphthalmia-associated transcription factor (MITF)-Tfe transcription factors were blocked using either a pan MITF-Tfe dominant negative or specific small interfering RNA (siRNA).

**Results:**

*VEGF* mRNA and protein secretion increased over time in the RPE cells cultured on Transwells, with protein secretion occurring in a polarized fashion primarily toward the basolateral side. Overexpression of a dominant negative that targets the MITF-Tfe family resulted in a 50% reduction in *VEGF* expression. The role of the MITF-Tfe family in *VEGF* regulation in the RPE was corroborated in studies with the *VEGF*-luciferase reporter constructs, where deletion of the distal *VEGF* promoter region containing putative binding sites for the MITF-Tfe family resulted in a 50% reduction in *VEGF* promoter activity. siRNA knockdown of the MITF-Tfe family individually, and in combination, revealed that downregulation of Tfe3 resulted in reduced *VEGF* expression.

**Conclusions:**

Our results indicate that Tfe3, in conjunction with other MITF-Tfe members, regulates *VEGF* expression in the RPE and are consistent with the hypothesis that *VEGF* expression in RPE cells is regulated as part of their differentiation.

## Introduction

Vascular endothelial growth factor (VEGF), also called VEGF-A, is a member of the cysteine-knot superfamily of growth factors, which is characterized by the presence of eight conserved cysteine residues [1,2]. VEGF has been shown to be an important regulator of normal and pathological angiogenesis, the process by which new blood vessels arise from existing ones [3]. VEGF also plays a role in controlling vascular permeability [4]. Targeted disruption of VEGF and its receptors results in early embryonic lethality due to severe defects in vasculogenesis [5–7]. VEGF expression declines postnatally, but continues in specific cells in virtually all tissues, particularly at sites proximal to fenestrated endothelium, such as podocytes adjacent to glomerular endothelium, and retinal pigment epithelium (RPE) adjacent to the choriocapillaris [8,9].

Our laboratory has shown that the RPE is the only source of VEGF in the back of the eye [10]. RPE-derived VEGF not only functions in the development [11], maintenance, and survival of the choriocapillaris and the RPE but also contributes to their pathologies. Aberrant VEGF expression by the RPE is believed to promote the progression of the choroidal neovascularization associated with the advanced form of age-related macular degeneration (AMD). However, little is known about the molecular regulation of VEGF in the RPE. Previous studies have identified various factors and conditions that are capable of influencing VEGF expression, including hypoxia, mechanical stress, advanced glycation end products, vasopressor hormones (angiotensin II, vasopressin), cytokines (interleukin 1 and tumor necrosis factor), and growth factors (TGFβ2, basic fibroblast growth factor, and platelet-derived growth factor) [12]. Patterns of VEGF expression observed in adults [13] have led to the hypothesis that cell-specific VEGF expression may be regulated as part of the process of cell differentiation, and our laboratory demonstrated that VEGF expression by skeletal muscle is regulated in coordination with myogenic differentiation [14]. Motivated by these observations, we aimed the current study at determining whether VEGF expression is also regulated in concert with RPE differentiation.

Transcriptional regulation of *VEGF* occurs through the core promoter as well as through enhancers or repressors outside the core promoter domain. *VEGF* is unique in that its core promoter does not contain typical transcriptional initiation recognition sequences, such as a TATA box or a transcription factor II B recognition element [15]. Researchers have predicted that the *VEGF* promoter is controlled by an SP1 site located 50 base pairs upstream of the transcription start site [15,16]. Enhancement or repression of *VEGF* basal transcription can be controlled by the interaction of specific transcription factors with the *VEGF* promoter through, or independent of, the basal transcriptional machinery.

The key transcription factors involved in RPE specification include Pax6, Otx2, and microphthalmia-associated transcription factor (MITF). Pax6 is considered the master regulator of eye development, as the absence of this factor leads to complete failure of eye development, known as anophthalmia [17,18]. Pax6 expression ultimately becomes restricted to the primitive retina, whereas Otx2 and MITF are localized in the primitive RPE [19,20]. MITF is the key RPE-specifying transcription factor. Inactivation of MITF in mouse or quail impairs the development of the presumptive RPE; the RPE remains non-pigmented and hyperproliferates, becoming an additional neural retina [21]. Otx2 is thought to act in conjunction with MITF in a feedback loop to control RPE-specific gene expression. Otx2 overexpression in retinal cells leads to pigmentation; the presumptive RPE of mice deficient in Otx1/Otx2 is almost completely devoid of MITF expression, lacks pigmentation, and displays an RPE-to-retina transdifferentiation [20,22]. MITF belongs to the MITF-Tfe family of transcription factors, which are basic helix–loop–helix-leucine zipper transcription factors, including MITF, Tfeb, Tfe3, and Tfec [23]. Therefore, to test the hypothesis that VEGF regulation is coordinated with cell differentiation, we evaluated the MITF-Tfe family as candidate modulators of *VEGF* expression in the RPE.

## Methods

### Cell culture, constructs, and transient transfections

ARPE-19, a human retinal pigment epithelium cell line, were purchased from ATCC (Mannassa, VA). Cells were cultured in Dulbecco’s modified Eagle’s medium (DMEM)/F12 1:1 supplemented with 10% fetal bovine serum (FBS) and 1% GlutaMAX, penicillin/streptomycin (GPS), and used through a maximum of 30 passages. ARPE-19 cells were induced to polarize by plating at high density (1.7×10^5^ cells) on laminin-coated 0.4 μm-pore 12-well Costar Transwells (Thermo Fisher Scientific, Cambridge, MA), and maintained in DMEM/F12 supplemented with 1% FBS and 1% GPS. Media were changed twice a week for 4 weeks before use in experiments. Human embryonic kidney 293 (HEK 293) cells (ATCC) were maintained at subconfluence in DMEM (Gibco; Rockville, MD) supplemented with 10% FBS and 1% GPS. Both cell types were incubated at 37 °C in a 5% CO_2_ incubator and subcultured using trypsin.

For transfection with dominant negative MITF (kindly provided by David Fisher, Harvard Medical School, Boston, MA), which targets the MITF-Tfe transcription factor family, the ARPE-19 media were changed to 0.1% serum the day before transfection and maintained in low serum conditions throughout the experiment to minimize the effect of serum on *VEGF* expression. ARPE-19 cells were transfected at 65%–75% confluence using Lipofectamine LTX with PLUS reagent (Invitrogen, Carlsbad, CA) in 0.1% FBS DMEM/F12 for 24 h, and then fresh medium containing 0.1% FBS DMEM/F12 and GPS was added. At 48 h post-transfection, cells were washed in phosphate buffer solution (PBS; 137 mM NaCl; 2.7 mM KCl; 4.3 mM Na_2_HPO_4_; 1.47 mM KH_2_PO_4_, pH of 7.4; Sigma-Aldrich, Saint Louis, MO), and RNA was isolated to analyze gene expression with real-time PCR (qRT–PCR).

### Immunofluorescence

Occludin was localized in polarized ARPE-19 cells. Cells were washed with PBS and fixed in ice-cold 4% paraformaldehyde for 10 min. After three washes in PBS, samples were blocked in 0.1% Triton/5% goat serum in PBS for 2 h at room temperature, and incubated with the occludin antibody (1:100; Invitrogen) overnight at 4 °C. After being washed with PBS, the samples were incubated with a mixture of fluorescein isothiocyanate (FITC)-conjugated goat antirabbit fluorescent secondary antibody (1:300; Jackson ImmunoResearch Laboratories; West Grove, PA) and 4',6-diamidino-2-phenylindole (DAPI; 1:100) for 1 h at room temperature. The Transwell membranes were cut around the periphery of the membrane with a razor blade, and then removed from the well with tweezers. The membranes were mounted on slides using aqueous mounting media consisting of 50% glycerol in PBS, and images were taken with an Axioskop (Axioskop MOT 2; Carl Zeiss Meditec, Inc., Dublin, CA).

### Small interfering RNA–mediated gene silencing

Knockdown of MITF, Tfeb, and Tfe3 was achieved with 0.2 μM of ON-TARGETplus SMARTpools containing four pooled siRNA duplexes (Dharmacon; Lafayette, CO). A scrambled siRNA pool (0.2 μM) that lacks identity with known gene targets was used to control for non-sequence-specific effects. ARPE-19 cells were transfected as above, and three time points (24, 48, 72 h) were tested to determine the optimal post-transfection time to isolate mRNA. Of the three times, maximal gene downregulation was noted at 48 h and was therefore selected as the time point at which mRNA was isolated for analysis with qRT–PCR.

### Luciferase reporter assays

ARPE-19 and HEK 293 cells at 75%–80% confluence were cotransfected using Lipofectamine 2000 (Invitrogen) and FuGENE 6 (Roche Diagnostics, Indianapolis, IN), respectively, with equal amounts of VEGF *firefly* luciferase (*F*luc)-reporter plasmid, and *Renilla* control luciferase (*R*luc; Promega, Madison, WI). The cells were transfected overnight, and fresh medium was added the following day. Lysates were collected 48 h post-transfection, and luciferase expression was detected using the Dual-Luciferase Reporter Assay System (Promega) with a Turner Luminometer (Turner Designs, Sunnyvale, CA), according to the manufacturer’s instructions. *VEGF* promoter activity, as indicated by the *F*luc activity, was normalized to *R*luc. The ratio of *F*luc to *R*luc was then divided by the ratio of the promoterless luciferase to determine the fold change.

### Real-time polymerase chain reaction analysis

Total mRNA was purified using RNA-Bee solution (Iso-Tex Diagnostic, Inc., Friendswood, TX) under RNase-free conditions, according to the manufacturer’s instructions. One microgram of RNA was reverse-transcribed using SuperScript III (Invitrogen), and cDNA that was diluted 1:20 (50 ng of equivalent RNA) was used in each amplification reaction. Reactions were performed using the SYBR Green Master Mix and the ABI Prism 9700 Sequence Detection System (Applied Biosystems, Carlsbad, CA) according to the manufacturer’s instructions. For primer sequences, see Table 1. The PCR cycles consisted of an initial denaturation step at 95 °C for 10 min, followed by 40 cycles at 95 °C for 15 s and at 60 °C for 60 s. Each sample was also subjected to melting curve analysis to confirm amplification specificity. Samples were run in duplicate, and each experiment included two non-template control wells. Samples were normalized to glyceraldehyde 3-phosphate dehydrogenase (*GAPDH*), and expressed as the relative expression using the delta-delta Ct method. Results are expressed as the mean±standard deviation.

**Table 1:** Real-time PCR primer sequences for human genes.

**Primer name**	**Forward sequence**	**Reverse sequence**
*GAPDH*	CAAATTCCATGGCACCGTCA	GGAGTGGGTGTCGCTGTTGA
*MITF*	AGCCATGCAGTCCGAAT	ACTGCTGCTCTTCAGCG
*Tfe3*	TCCTGAAGGCCTCTGTGGAT	AGGTCCAGAAGGGCATCTGA
*Tfeb*	CGC ATCAAGGAGTTGGGAAT	CTCCAGGCGGCGAGAGT
*TRP-1*	TCTCTGGGCTGTATCTTCTTCC	GGCAACACATACCACTTCTCAA
Tyrosinase	CATTCTTCTCCTCTTGGCAGA	CCGCTATCCCAGTAAGTGGA
*VEGF*	ATCGAGACCCTGGTGGACA	CCGCCTCGGCTTGTCACA

### Enzyme-linked immunosorbent assay for determining vascular endothelial growth factor levels

Conditioned media were collected from the apical and basolateral compartments of ARPE-19 cells that were polarized over the course of 4 weeks. On the day before the conditioned media were collected, the media were changed so that the levels of secreted VEGF detected would reflect the amount of VEGF secreted over the course of 24 h. The difference in volume of the apical (500 μl) versus the basolateral (1.5 ml) compartment was accounted for in the final calculation. VEGF levels were determined with enzyme-linked immunosorbent assay (ELISA; R&D Systems, Minneapolis, MN), according to the manufacturer’s protocol.

### Statistical analysis

Values are expressed as the mean±standard deviation, unless otherwise indicated. Statistical analysis was performed using an unpaired Student *t* test (***: p<0.001, **: p<0.01, *: p<0.05, ns: p>0.05).

## Results

### *VEGF* expression in the retinal pigment epithelium increased during in vitro differentiation

**Figure 1:**
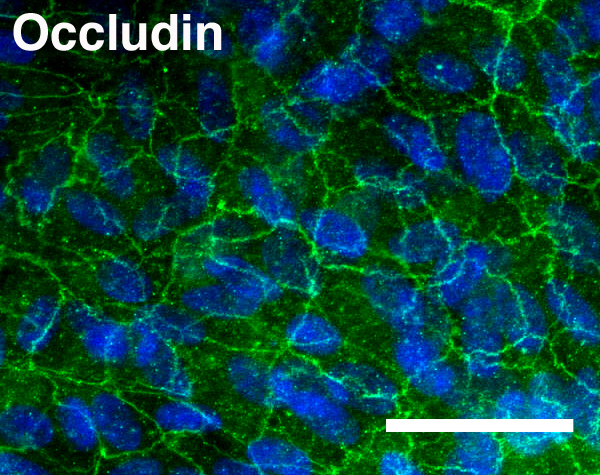
Occludin localization in polarized human retinal pigment epithelium (ARPE-19) cells. Occludin (green) and 4',6-diamidino-2-phenylindole (DAPI; blue) immunolocalization in ARPE-19 cells that were cultured for 4 weeks on Transwells to induce polarization reveals circumferential junctions and demonstrates a cuboidal monolayer. Scale bar is 50 μm.

**Figure 2:**
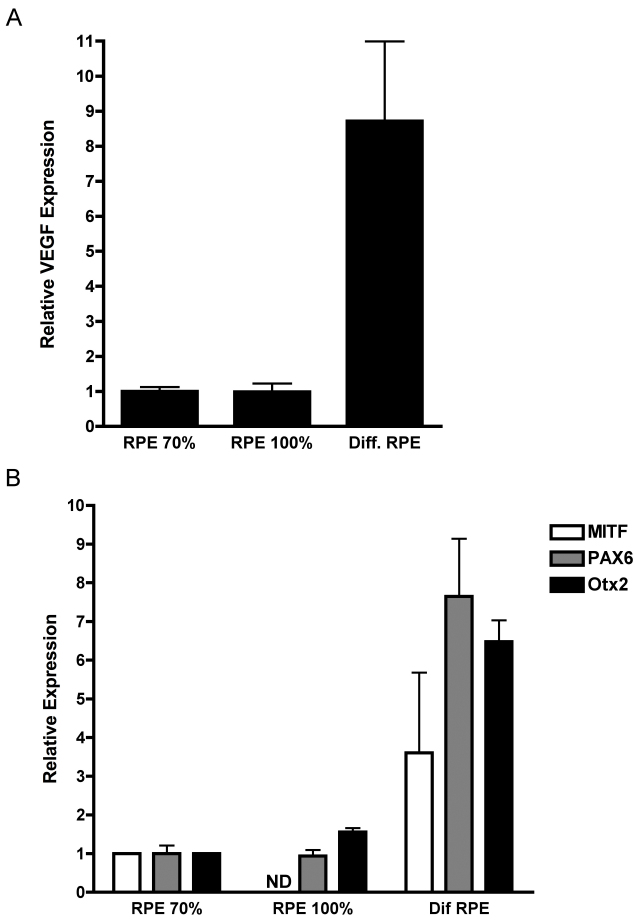
Vascular endothelial growth factor (VEGF) and retinal pigment epithelium (RPE) transcription factor expression during RPE in vitro maturation. qRT–PCR analysis was performed on cDNA isolated from human retinal pigment epithelium (ARPE-19) cells that were maximally differentiated for 6 weeks and cells cultured on plastic that were 70% or 100% confluent. (**A**) VEGF and (**B**) RPE-transcription factors were upregulated in polarized ARPE-19 cells compared to non-polarized cells cultured on plastic. The experiment was performed in triplicate, and the results are expressed as the mean±standard deviation.

**Figure 3:**
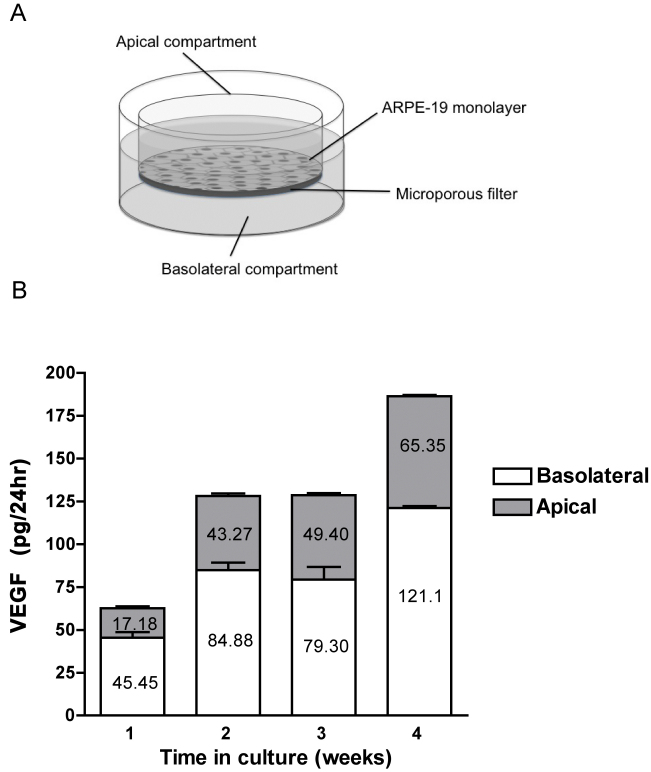
Retinal pigment epithelium (RPE) secretion of vascular endothelial growth factor (VEGF) during in vitro polarization. Conditioned media were collected from the apical and basolateral Transwell compartments over the course of 4 weeks and analyzed for VEGF with enzyme-linked immunosorbent assay (ELISA). **A**: Diagram illustrating Transwell system used for culture of human retinal pigment epithelium (ARPE-19) cells. **B**: An increase in VEGF secretion preferentially toward the basolateral side was observed during the 4-week culturing. The experiment was performed in triplicate, and the results are expressed as the mean±standard deviation.

As the first step in elucidating the molecular regulation of *VEGF* in the RPE, *VEGF* expression was analyzed in ARPE-19 cells in an in vitro model of cell maturation. ARPE-19 cells are an immortalized human RPE cell line, a useful tool for studying RPE cells in vitro. ARPE-19 cells recapitulate many characteristics of RPE in vivo, including the distribution of VEGF isoform expression [24], the formation of tight junctions [24,25], and increased transepithelial resistance [25]. The in vitro model of maturation consists of culturing cells in low serum on laminin-coated Transwell membranes; ARPE-19 cells mature rapidly over 4 weeks and continue to mature slowly thereafter [26]. Localization of occludin, a tight junction marker, demonstrated a cuboidal monolayer, which is associated with mature ARPE-19 cells (Figure 1). Compared to subconfluent (70%) or confluent cells on plastic, VEGF in ARPE-19 cells cultured for 6 weeks on Transwells was upregulated eightfold (Figure 2). These ARPE-19 cells also displayed increased expression of Pax6, MITF, and Otx2, the key RPE-specifying transcription factors (Figure 2). Evaluation of VEGF protein secretion during the 4 weeks of in vitro maturation revealed that VEGF secretion correspondingly increased over time, with approximately 189 pg/ml VEGF detected at week 4 compared to approximately 62 pg/ml at week 1. VEGF secretion was polarized, occurring primarily (65%–72%) toward the basolateral side of the RPE (Figure 3).

### Distal promoter regions are essential for *VEGF* expression in the retinal pigment epithelium

**Figure 4:**
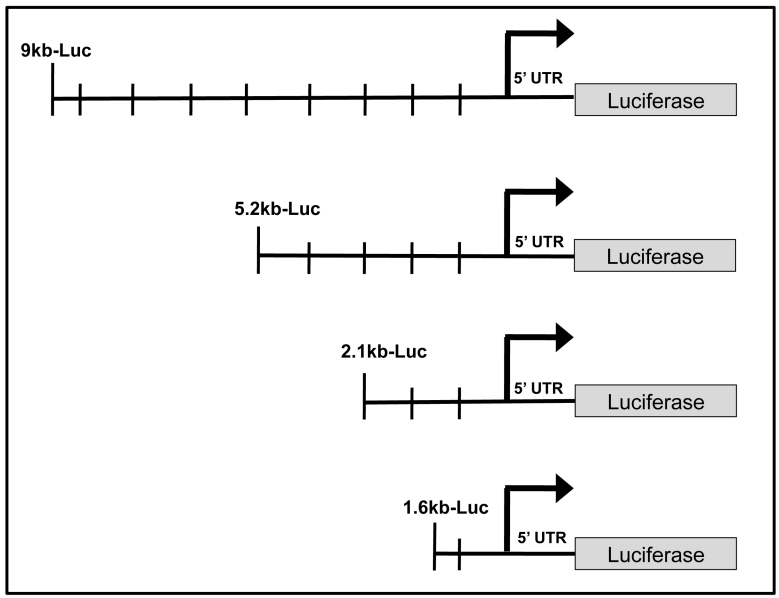
Schematic of vascular endothelial growth factor (*VEGF*)-luciferase reporter constructs. *VEGF*-luciferase constructs indicating the size of upstream *VEGF* promoter sequence, transcription start site, and 5′ UTR. *VEGF* genomic DNA was ligated upstream of a promoterless luciferase gene in the pGL2-basic plasmid. Deletions were made by taking advantage of the restriction sites located within the *VEGF* promoter and the pGL2 multiple cloning site [16].

**Figure 5:**
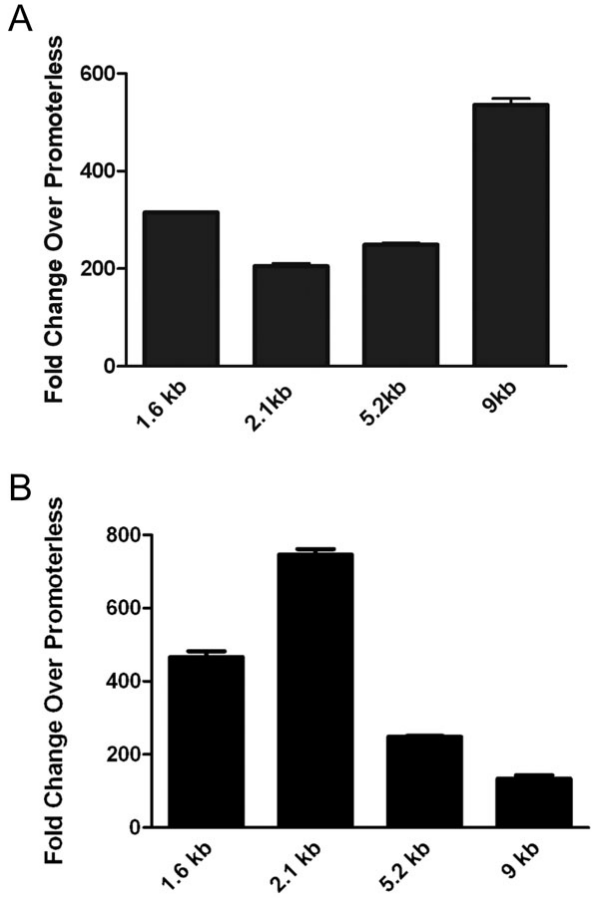
Vascular endothelial growth factor (*VEGF*) promoter regions involved in regulating *VEGF* expression in RPE. (**A**) Human retinal pigment epithelium cell line (ARPE-19) or (**B**) HEK 293 cells were transfected with *VEGF*-luciferase constructs containing various truncations of the *VEGF* promoter. There was a twofold induction of promoter activity with the 9 kb construct over the 5.2 kb construct in (**A**) ARPE-19 cells. In (**B**) HEK 293 cells, maximum promoter activity was achieved with the 2.1 kb construct. The experiment was performed in triplicate. A representative experiment is shown with data expressed as the mean±standard deviation.

**Figure 6:**
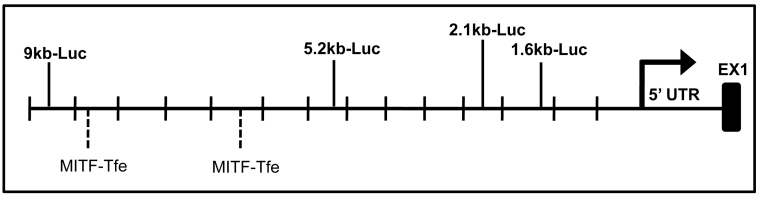
Schematic of putative binding sites for transcription factors in vascular endothelial growth factor (*VEGF*) promoter. The putative binding sites for the MITF-Tfe family are primarily concentrated in the -5 kb to -9 kb region of the *VEGF* promoter.

To begin to examine the molecular basis of *VEGF* expression, constructs containing various truncations of the *VEGF* promoter controlling luciferase were used to determine the regions of the *VEGF* promoter that are essential for *VEGF* expression in the RPE. These constructs consisted of a 9 kb full-length *VEGF* promoter, as well as deletion mutants containing 5.2 kb, 2.1 kb, and 1.6 kb of upstream *VEGF* promoter (Figure 4). Analysis of these constructs in ARPE-19 cells revealed a twofold induction of promoter activity with the full-length 9 kb *VEGF* reporter construct over the 5.2 kb construct. Interestingly, similar induction of promoter activity was not observed in a control cell type (HEK 293), in which maximal promoter activity was obtained with the 2.1 kb construct (Figure 5). This observation suggests that RPE may express transcription factors or enhancers that bind specifically in the distal region of the *VEGF* promoter. Sequence analysis of the *VEGF* promoter revealed numerous binding sites for several transcription factors required for RPE specification; most notably, the putative binding sites for the MITF-Tfe transcription factor family were concentrated primarily in the -5 kb to -9 kb region (Figure 6).

### MITF-Tfe family is involved in regulating *VEGF* in the retinal pigment epithelium

**Figure 7:**
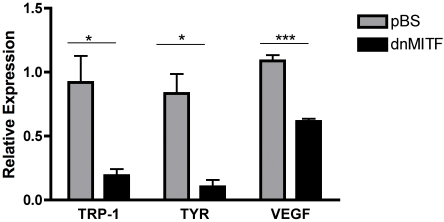
Overexpression of dnMITF leads to downregulation of vascular endothelial growth factor (*VEGF*) in the retinal pigment epithelium (RPE). Human retinal pigment epithelium (ARPE-19) cells were transfected with a dominant negative against the MITF-Tfe transcription factor family (dnMITF) or an empty vector control (pBS). RNA was isolated 48 h post-transfection and analyzed with qRT–PCR. A 50% reduction in *VEGF* mRNA was observed in samples overexpressing the dnMITF; there was an 80% decrease in the expression of known targets for the MITF-Tfe family, tyrosinase (TYR), and tyrosinase-related protein-1 (TRP-1). The experiment was performed in triplicate. A representative experiment is shown with data expressed as the mean±standard deviation. *: p<0.05; ***: p<0.001.

To determine if the transcription factors important for RPE specification might also be involved in regulating *VEGF* expression, the entire MITF-Tfe family was inhibited with dominant negative, or knocked down individually with siRNA. Overexpression of a dominant negative construct targeting the MITF-Tfe family led to a significant (50%) reduction in *VEGF* expression (Figure 7). The MITF-Tfe family of transcription factors are basic helix–loop–helix-leucine zipper transcription factors, including MITF, Tfeb, Tfe3, and Tfec [23]. These transcription factors may bind as homodimers or heterodimers to regulate gene transcription, making the pan MITF-Tfe dominant negative highly useful in determining the role of this transcription factor family in modulating gene expression. Notably, the downregulation of *VEGF* expression following pan MITF-Tfe blockade was seen only when transfections were performed in low serum (0.1%), indicating a role for serum factor in regulating *VEGF* expression. The extent of *VEGF* downregulation achieved with dominant negative pan MITF-Tfe closely paralleled that observed with the truncated 5.2 kb *VEGF*-luciferase reporter construct that lacks the region containing the binding sites for the MITF-Tfe family. Taken together, these findings support the notion that this family is involved in regulating *VEGF* expression, either directly or indirectly.

**Figure 8:**
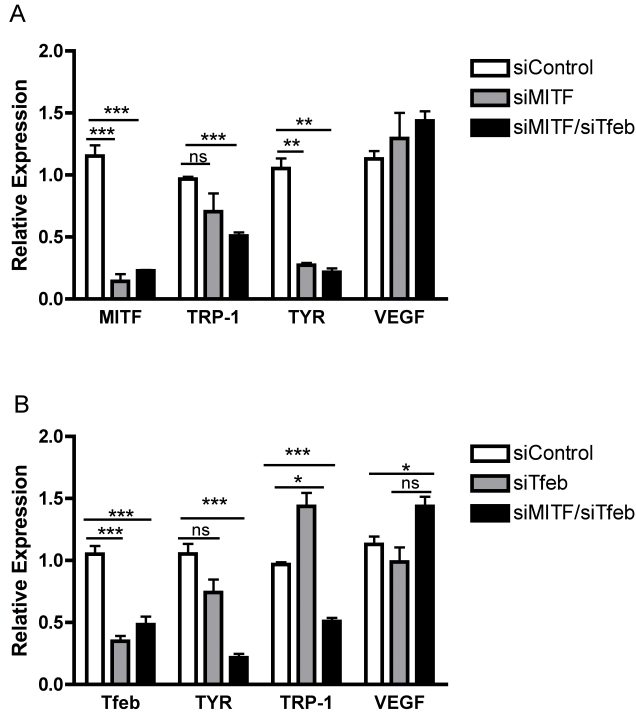
Knockdown of MITF and/or Tfeb does not affect vascular endothelial growth factor (*VEGF*) expression. Human retinal pigment epithelium (ARPE-19) cells were transfected with an siControl or a SMARTpool targeting MITF and/or Tfeb. Depletion of (**A**) MITF and/or (**B**) Tfeb did not reduce *VEGF* expression. The experiment was performed in triplicate. A representative experiment is shown with data expressed as the mean±standard deviation. *: p<0.05; ***: p<0.001; ns: p>0.05.

**Figure 9:**
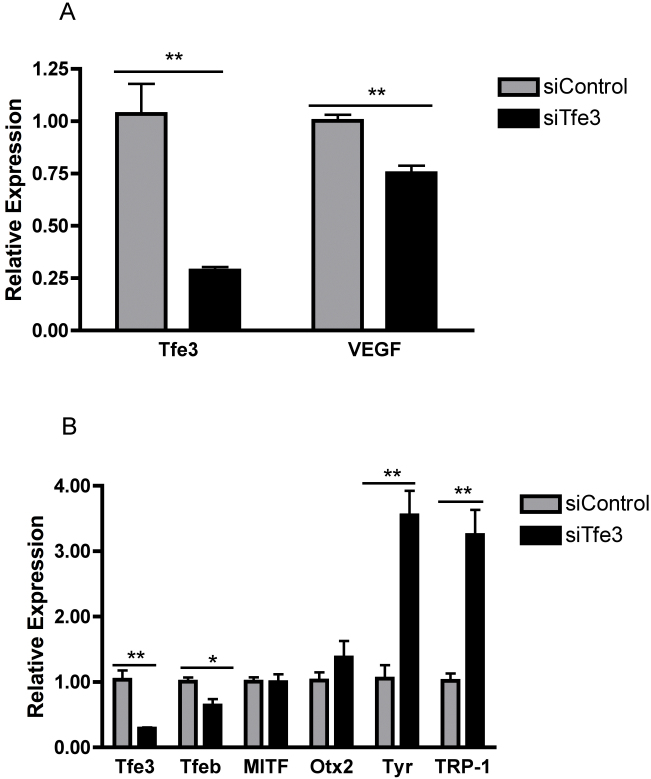
Tfe3 knockdown leads to a reduction in vascular endothelial growth factor (VEGF) expression. Human retinal pigment epithelium (ARPE-19) cells were transfected with an siControl or a SMARTpool targeting Tfe3. **A**: A 20% reduction in VEGF expression and (**B**) a three- to fourfold increase in Tfe3 downstream targets TRP-1 and tyrosinase were observed with Tfe3 depletion. The experiment was performed in triplicate. A representative experiment is shown with data expressed as the mean±standard deviation. *: p<0.05; **: p<0.01.

To determine which member(s) of the MITF-Tfe family may mediate *VEGF* regulation, pooled siRNA against MITF and Tfeb was used. MITF and Tfeb were successfully knocked down, individually and in combination, yet no effect of their knockdown on *VEGF* expression was observed (Figure 8). Tfe3 knockdown with siRNA led to a 20% reduction in *VEGF* mRNA; however, there was also a three- to fourfold increase in the known target genes of *Tfe3* (tyrosinase-related protein 1 and tyrosinase; Figure 9). Although the knockdown of MITF, Tfeb, and Tfe3 was significant at 60%–80%, there is a possibility that even a low level of expression of these factors may be sufficient to influence *VEGF* expression in the RPE. Alternatively, there could be a redundancy among the family members so that inhibiting all members of the family would be necessary to affect *VEGF* expression. Nevertheless, these data provide compelling support for the concept that members of this family of transcription factors, particularly Tfe3, are involved in regulating *VEGF* expression in the RPE.

## Discussion

Analysis of ARPE-19 cells in vitro revealed that *VEGF* mRNA and protein secretion increased during culture for 4 to 6 weeks on Transwells during which time the cells matured rapidly. VEGF secretion by ARPE-19 may continue to increase during longer-term in vitro maturation (>1 month), but we also suspect that the secretion would eventually plateau. We found that VEGF protein secretion was also polarized, occurring primarily toward the basolateral side, which is consistent with the physiologic evidence that RPE-derived VEGF supports the underlying choriocapillaris. The preferential secretion of VEGF toward the basolateral side corroborated previous findings in primary human RPE cells [27]. Alternatively, another report suggested that the secretion may not be polarized, but rather that a higher rate of VEGF degradation and transcytosis at the apical surface of RPE cells accounts for the difference [28]. Nevertheless, the fact that the RPE uses such mechanisms further supports the physiologic importance of controlling the amount of VEGF secreted apically to retain an avascular outer retina. Given the role of VEGF as a potent angiogenic factor, it has been proposed that the outer retina is able to remain avascular by complementary apical secretion of the anti-angiogenic factor pigment epithelium-derived factor (PEDF) by the RPE. A recent study reported that PEDF secretion also increases in phenotypically polarized human embryonic stem cell-derived RPE [29]. Thus, a balance between angiogenic and antiangiogenic factor secretion is required to maintain eye homeostasis during development, and in the adult.

VEGF expression occurs early during eye development, at embryonic day 9.5 [24] when RPE specification is occurring. This observation combined with the increased VEGF expression during RPE maturation further supports the notion of coordinated regulation of VEGF expression and cell differentiation. VEGF functions to control tissue vascularization during development [5,30], and maintains the vasculature in the adult [31–33]. In light of these critical roles, it is not surprising that regulation of VEGF in the RPE might be synchronized with cell differentiation.

To directly test the hypothesis of regulation of VEGF coordinated with cell differentiation, the role of transcription factors involved in RPE specification in regulating VEGF expression was examined. Use of the pan MITF-Tfe dominant negative provided strong evidence that the MITF-Tfe family of transcription factors regulates VEGF expression in the RPE. This observation was supported by the fact that the distal region of the VEGF promoter, which contains putative binding sites for the MITF-Tfe family, was essential for expression by RPE, but not by HEK 293 cells. The fact that 50% was the maximum reduction of VEGF expression achieved using the pan MITF-Tfe dominant negative suggests that VEGF expression by the RPE is mediated by multiple mechanisms. The reduction of VEGF expression by the dominant negative MITF-Tfe was detected in 0.1% serum, and the remaining 50% of VEGF expression is likely due, at least in part, to serum components, which have been shown to induce expression of VEGF family members [34].

Investigation of the relative contribution of individual MITF-Tfe family members revealed that of the family members tested, only knockdown of Tfe3 produced a reduction in VEGF expression. Interestingly, the reduction of VEGF expression occurred without a decrease in Tfe3 known targets. This observation is likely because alternative splicing of Tfe3 gives rise to two isoforms: Tfe3L and Tfe3S. Tfe3S, which lacks the N-terminal acidic activation domain present in Tfe3L, has a fourfold lower activation potential than Tfe3L, and has been reported to have a dominant negative effect against Tfe3L [35]. Therefore, knockdown of Tfe3S would eliminate its dominant negative effect and may well account for the increased expression of the downstream targets, tyrosinase-related protein 1 and tyrosinase. Given that depletion of the entire MITF-Tfe family was necessary to affect a 50% reduction in VEGF expression indicates functional redundancy among the members of the MITF-Tfe family.

The pattern and timing of VEGF expression in vivo are consistent with a role for the MITF-Tfe family in modulating VEGF expression by the RPE. MITF expression commences in the optic vesicle at E9.0, and by E9.5 MITF is localized primarily in the proximal optic vesicle, with less expression in the region of the optic vesicle adjacent to the surface ectoderm [21]. VEGF expression in the developing retina arises between E9.0 and E9.5, and at E9.5, VEGF is primarily detected in the proximal optic vesicle [24]. At E9.5, MITF and VEGF have similar patterns of expression. In addition, the Tfeb knockout mouse is embryonic lethal, with mice dying between E9.5 and E10.5 due to placental vascularization defects [36]. Interestingly, labyrinthine cells in the mutant Tfeb placenta failed to express VEGF, suggesting that Tfeb regulates VEGF in the placenta. Taken together with our findings, it appears that Tfe3, in conjunction with other MITF-Tfe members, may regulate VEGF expression in the RPE.

There is increasing evidence for a link between the development and progression of AMD and the status of the RPE. A recent report illustrated that oxidative stress can lead to RPE dedifferentiation and hypertrophy, which leads to decreased expression of several essential genes involved in RPE functions, including phagocytosis (MER tyrosine kinase), and the conversion of all-trans-retinal to 11-cis-retinal (RPE65) [37]. Interestingly, of the six RPE-characteristic markers tested in that study, only MITF increased in expression. The dysfunctional RPE may lead to photoreceptor cell death, while the elevated MITF could cause increased VEGF expression, promoting progression to the advanced form of AMD.
